# *Mycobacterium riyadhense* in Saudi Arabia

**DOI:** 10.3201/eid2310.161430

**Published:** 2017-10

**Authors:** Bright Varghese, Mushirah Abdulaziz Enani, Sahar Althawadi, Sameera Johani, Grace Mary Fernandez, Hawra Al-Ghafli, Sahal Al-Hajoj

**Affiliations:** King Faisal Specialist Hospital and Research Centre, Riyadh, Saudi Arabia (B. Varghese, S. Althawadi, G.M. Fernandez, H. Al-Ghafli, S. Al-Hajoj);; King Fahad Medical City, Riyadh (M.A. Enani);; King Abdul Aziz Medical City, Riyadh (S. Johani)

**Keywords:** *Mycobacterium riyadhense*, Saudi Arabia, nontuberculous mycobacteria, tuberculosis, bacteria

## Abstract

We explored in detail the nationwide existence of *Mycobacterium riyadhense* in Saudi Arabia. In 18 months, 12 new cases of *M. riyadhense* infection were observed, predominantly among Saudi nationals, as a cause of pulmonary disease. *M. riyadhense* may be emerging as a more common pathogen in Saudi Arabia.

Infections caused by nontuberculous mycobacteria (NTM) appear to be emerging globally, but the definitive reasons for this are unclear. Advances in diagnostic technologies have led to the identification of >160 species of *Mycobacterium*, including several human pathogens. *M. riyadhense* is a slow-growing NTM identified as a cause of pulmonary and extrapulmonary illnesses in humans from Riyadh, Saudi Arabia (*1*,*2*). At least 8 clinical cases have been reported from France, Bahrain, Saudi Arabia, and South Korea, with 5 of the 8 cases in Saudi Arabia (*1*–*6*) (Table). *M. riyadhense* can be misidentified by commercially available line probe assays as *M. tuberculosis* complex, mostly because of confusing banding patterns (*1*). A recent nationwide study of NTM prevalence in Saudi Arabia showed no suspected cases of *M. riyadhense*, which could be due to limiting the screening to line probe assays (*7*).

**Table:** Summary of all reported *Mycobacterium riyadhense* infections in Saudi Arabia and other countries*

**Case**	**Age, y/sex**	**Nationality**	**City**	**Region/ country**	**Specimen**	**Smear/ culture results**	**Clinical relevance†**	**Treatment†**		**Treatment outcome**
**This study**										
** 1**	25/M	Saudi	Dammam	Eastern/ Saudi Arabia	Sputum	++/+	Yes	CLR/INH/RFP	Cured	
** 2**	55/M	Saudi	Riyadh	Central/ Saudi Arabia	BAL	–/+	Yes	INH/RFP/EMB/PZA	Cured	
** 3**	39/F	Non-Saudi	Riyadh	Central/ Saudi Arabia	Sputum	+/+	Yes	INH/RFP/EMB/PZA	Cured	
** 4**	77/M	Saudi	Riyadh	Central/ Saudi Arabia	Tracheal aspirate	+/+	Yes	INH/RFP	Cured	
** 5**	57/M	Saudi	Riyadh	Central/ Saudi Arabia	Lymph node	–/+	Yes	INH/RFP/CLR	Cured	
** 6**	82/M	Saudi	Riyadh	Central/ Saudi Arabia	BAL	–/+	Yes	CLR/INH/RFP	Cured	
** 7**	18/M	Saudi	Riyadh	Central/ Saudi Arabia	Gastric aspirate	+/+	Yes	INH/RFP/EMB/PZA	Cured	
** 8**	32/M	Non-Saudi	Riyadh	Central/ Saudi Arabia	Endotracheal aspirate	–/+	Yes	CLR/INH/RFP	Cured	
** 9**	61/M	Saudi	Riyadh	Central/ Saudi Arabia	Sputum	+/+	Yes	INH/RFP	NA	
** 10**	8/M	Saudi	Riyadh	Central/ Saudi Arabia	Lymph node	–/+	Yes	CLR/INH/RFP	Cured	
** 11**	82/M	Saudi	Dammam	Eastern/ Saudi Arabia	Sputum	+/+	No	INH/RFP	Died	
** 12**	28/M	Saudi	Riyadh	Central/ Saudi Arabia	Lymph node	–/+	Yes	INH/RFP	Cured	
**Previous reports**										
** (** ***1*** **)**	19/M	Saudi	Riyadh	Central/ Saudi Arabia	Bone infection in maxillary sinus	–/+	Yes	INH/RIF/EMB/PZA	Cured	
** (** ***2*** **)**	38/F	South Korea	NA	South Korea	Sputum	+/+	Yes	INH/RIF/EMB/PZA	Cured	
** (** ***3*** **)**	39/F	France	Toulon	France	Sputum	+/+	Yes	INH/RIF/EMB/PZA	Cured	
** (** ***4*** **)**	43/M	Bahrain	Awali	Bahrain	Sputum	–/+	Yes	INH/RIF/EMB/PZA/CLR/CIP	Cured	
** (** ***5*** **)**	18/F	Saudi	Jeddah	West/ Saudi Arabia	Brain with bone	–/+	Yes	INH/RIF/EMB/PZA/MX	Cured	
** (** ***6*** **)**	24/F	Saudi	Riyadh	Central/ Saudi Arabia	Spine	–/+	Yes	INH/RIF/EMB/PZA	Cured	
** (** ***7*** **)**	30/M	Saudi	NA	West/ Saudi Arabia	Sputum + lymph node	+/+	Yes	INH/RIF/EMB/PZA	Cured	
** (** ***8*** **)**	54/M	Saudi	NA	Central/ Saudi Arabia	BAL	+/+	Yes	INH/RIF/EMB/PZA	Cured	

*BA, bronchoalveolar lavage; NA, not available; +, positive; -, negative. Positive smearing results indicate the presence of acid-fast bacilli (AFB). Wherein, 10–99 AFB identified in 100 fields have been noted with (+), and 100–999 AFB in 100 fields correlates with (++). Positive culturing results highlight the presence of mycobacterial growth. CIP, ciprofloxacin; CLR, clarithromycin; EMB, ethambutol; INH, isoniazid; MX, moxifloxacin; PZA, pyrazinamide; RFP, rifampin.  **†Based on American Thoracic Society guidelines for pulmonary NTM disease/colonization (https://www.thoracic.org/statements/resources/mtpi/nontuberculous-mycobacterial-diseases.pdf).**

To explore the presence of *M. riyadhense* in clinical settings in Saudi Arabia, we conducted a prospective study on a nationwide collection of isolates. Suspected NTM isolates reported as *M. tuberculosis complex* or *Mycobacterium* species with nonspecific banding pattern by line probe assays were subjected to different conservative gene sequencing to identify *M. riyadhense.*

During April 2014–September 2015, we collected 458 NTM isolates, with clinical and epidemiological data, from all 9 national referral laboratories in different provinces of Saudi Arabia. We formulated the isolate enrollment strategy to suspect *M. riyadhense* on the basis of previous studies (*1*,*2*). In brief, we conducted primary identification of the isolates using line probe assay-Genotype MTBC (Hain Lifescience, Nehren, Germany). We further tested isolates that showed a nonspecific banding pattern (1,2,3) by using Genotype Mycobacteria CM and AS assays (Hain Lifescience). The Genotype Mycobacteria CM assay showed a specific banding pattern of 1,2,3,10,15,16 (1,2,3,10,16 in previous study) for a group of isolates; AS assay identified these isolates as *Mycobacterium* species. We subjected all isolates to partial sequencing of 16S rRNA, *rpoB* and *hsp65* genes using BigDye Terminator chemistry (Applied Biosystems, Foster City, CA, USA) (*8*–*10*). We then subjected the assembled sequences of all 3 genes to analysis via BLAST (https://blast.ncbi.nlm.nih.gov) and the EzTaxon database. We followed stringent identification criteria, requiring similarity >99% between isolate and reference strain for species confirmation.

We identified 14 isolates that fit the inclusion criteria; most were reported from the Central province, Riyadh, in Saudi Arabia, but the reason is unclear. Microbiological analysis showed slow-growing mycobacteria producing rough white colonies on LJ medium within 3–4 weeks of incubation at 37°C. Primary sequencing of the 16S rRNA gene showed 12 cases of *M. riyadhense* had a 99%–100% match with 3 database strains (GenBank accession nos. JF896094, JF896095, and NR044449). On the other hand, *rpoB* and *hsp65* sequences also showed 99%–100% similarity with other sequences (accession nos. EU921671, EU27644.1, JF86095 and NR 04449.1). The other closest species observed during the analysis were *M. alsense*, *M. szulgai*, and *M. angelicum* (98% similarity with 16S rRNA gene sequences); *M. genavens* and *M. simulans* (96% similarity with *hsp65* gene sequences); and *M. lacus*, *M. intracellulare*, and *M. malmoense* (94% similarity with *rpoB* gene sequences). Two isolates that matched inclusion criteria could not be identified as *M. riyadhense*; BLAST analysis showed the closest matching species as *M. lacus* DSM 44577(T), with 89% similarity. Two 16S rRNA gene sequences from this study were deposited in GenBank (accession nos. KX898970 and KX898971).

We identified 12 clinical cases of *M. riyadhense* infection, including pulmonary and extrapulmonary invasive infections, over a period of 18 months. Demographically, Saudi citizens dominated; 11 of 12 case-patients were male, and mean age was 50 years. Geographic distribution of cases showed 10 cases from Riyadh (Central province) and 2 from Dammam (Eastern province). Clinical data revealed 9 cases with pulmonary involvement and 3, including a pediatric case, with lymphadenitis. Of note, 75% of the respiratory cases were clinically relevant according to American Thoracic Society criteria for NTM pulmonary disease. Most patients recovered with isoniazid, rifampin, and ethambutol therapy (Table).

The lack of advanced molecular diagnostic tools in clinical laboratories in Saudi Arabia impedes the accurate identification of *M. riyadhense*. Without an accurate diagnosis, treatment is delayed. In this study, most of the patients were treated with standard TB regimens; some of them received clarithromycin, which did not appear to be highly effective (*2*). To date, no standard treatment regimen for *M. riyadhense* disease has been developed, likely due to its status as a rare species. In the cases reported here, patients generally responded well to the initial therapies, but drug resistance may challenge the empirical treatment used. A strain resistant to isoniazid is already reported from South Korea (*3*). We recommend that clinicians in Saudi Arabia be vigilant to the possible emergence of *M. riyadhense* as a more common pathogen.
